# Defining key metabolic roles in osmotic adjustment and ROS homeostasis in the recretohalophyte *Karelinia caspia* under salt stress

**DOI:** 10.1111/ppl.13663

**Published:** 2022-03-14

**Authors:** Qiang Guo, Jiwan Han, Cui Li, Xincun Hou, Chunqiao Zhao, Qinghai Wang, Juying Wu, Luis A. J. Mur

**Affiliations:** ^1^ Institute of Grassland, Flowers, and Ecology Beijing Academy of Agriculture and Forestry Sciences Beijing China; ^2^ College of Software Shanxi Agricultural University Taigu China; ^3^ Institute of Biological, Environmental, and Rural Sciences Aberystwyth University Aberystwyth UK

## Abstract

The recretohalophyte *Karelinia caspia* is of forage and medical value and can remediate saline soils. We here assess the contribution of primary/secondary metabolism to osmotic adjustment and ROS homeostasis in *Karelinia caspia* under salt stress using multi‐omic approaches. Computerized phenomic assessments, tests for cellular osmotic changes and lipid peroxidation indicated that salt treatment had no detectable physical effect on *K. caspia*. Metabolomic analysis indicated that amino acids, saccharides, organic acids, polyamine, phenolic acids, and vitamins accumulated significantly with salt treatment. Transcriptomic assessment identified differentially expressed genes closely linked to the changes in above primary/secondary metabolites under salt stress. In particular, shifts in carbohydrate metabolism (TCA cycle, starch and sucrose metabolism, glycolysis) as well as arginine and proline metabolism were observed to maintain a low osmotic potential. Chlorogenic acid/vitamin E biosynthesis was also enhanced, which would aid in ROS scavenging in the response of *K. caspia* to salt. Overall, our findings define key changes in primary/secondary metabolism that are coordinated to modulate the osmotic balance and ROS homeostasis to contribute to the salt tolerance of *K. caspia*.

## 1 INTRODUCTION

Salinization is a serious environmental problem in arid and semi‐arid regions, which restricts crops productivity and disrupts ecosystem functions (Tang et al., 2015; Yang et al., 2019a). Global food requirements are expected to increase by 70–110% by 2050 to meet the needs of the rapidly growing human population (Munns et al., 2012), but most crops are very sensitive to high salt, causing osmotic stress and disrupting ion homeostasis (Munns & Tester, 2008). However, halophytes have evolved various mechanisms to ensure their survival and to complete their life cycle in high salt environments (Flowers & Colmer, 2008). They can be used not only in the improvement of saline soils (remediation) but also a promising source of food, fiber, and medicine (Flowers & Muscolo, 2015; Song & Wang, 2014). Therefore, understanding the mechanisms employed by halophytes to adapt to environmental stresses could be important in breeding programs to improve the salt tolerance of crops (Cui et al., 2020).

The tolerance of plants to salt depends on ion homeostasis, synthesizing metabolites involved in osmotic adjustment and activating antioxidant defense systems (Isayenkov & Maathuis, 2019). One of the effective adaptive strategies in halophytes is the preferential transport of Na^+^ into the shoots, where it is compartmentalized into the vacuoles to maintain a low osmotic potential and ion homeostasis under high salinity (Guo et al., 2020a; Shabala, 2013). However, vacuoles become saturated if subjected to long‐term high salinity, resulting in the retention of Na^+^ in the cytoplasm, which affects the osmotic balance, inhibits metabolic processes and generates excessive ROS (reactive oxygen species; Flowers et al., 2015; Yuan et al., 2015). To counter this, elevated levels of primary metabolites (PMs) in the cytosol can aid to stabilize the internal osmotic potential, strengthen the thermodynamic stability of folded proteins, and protect macromolecular structures by reducing ROS accumulation (Slama et al., 2015; Yancey, 2005). For example, the very high salt tolerance of *Suaeda salsa* could be linked to the higher level of branched‐chain amino acids and saccharides in leaves compared with the less tolerant halophyte *Salicornia europaea* (Wang et al., 2021). In the halophyte *Limonium albuferae*, fructose and glucose accumulated, accompanied by a steady level of citric and malic acid, compared with the more salt sensitive *L. doufourii* (González‐Orenga et al., 2019). In recretohalophytes, salt is secreted from glands to improve salt tolerance. However, under high salt, recretohalophytes with epidermal bladder cells (EBC), such as *Chenopodium quinoa*, can accumulate more metabolites, including proline, γ‐aminobutyric acid (GABA) and inositol, to modulate ion transport than non‐EBC (Kiani‐Pouya et al., 2017).

Besides a role of PMs in salt responses, a significant contribution is made by secondary metabolites (SMs). Thus, such as tocopherol, phenolic acids and flavonoids, derived from the shikimate pathway, can contribute as nonenzymatic antioxidants (Muñoz & Munné‐Bosch, 2019; Sirin & Aslım, 2019). For example, the overexpression of *γ‐TMT* (*γ‐TOCOPHEROL METHYLTRANSFERASE*), gene coding for a key enzyme of tocopherol biosynthesis, increased the α‐tocopherol (vitamin E) content, hence contributing to decreased ROS, lipid peroxidation and ion leakage, conferring salt tolerance to the transgenic tobacco (Jin & Daniell, 2014). In honeysuckle (*Lonicera japonica*), the accumulation of leaf phenolic acids, including chlorogenic acid and luteolosid, suppressed the overproduction of ROS upon salt treatment (Yan et al., 2017). Similarly, β‐carotene, vitamin C, phenolic acids, and flavonoids were significantly increased by salt treatment, and acted as ROS scavengers in *Amaranthus tricolor* (Sarker & Oba, 2018). Such observations indicate a coordinated action of PMs and SMs as osmoprotectants and ROS scavengers to balance the osmotic pressure and control ROS overproduction when plants are subjected to salt stress. However, little is known about these mechanisms in the salt tolerance of recretohalophytes.

*Karelinia caspia* is a recretohalophyte, perennial herb Asteraceae mainly distributed in semi‐desert areas and desert grassland in northwestern China and shows strong adaptability to salinity, drought, and high temperature (Zhang et al., 2014). It is not only an essential forage species for livestock in desert grasslands, but also a pioneer species that plays a crucial role in the improvement of saline soils and desertification control (Wang et al., 2013; Zhang et al., 2020). A previous study showed that the tonoplast Na^+^/H^+^ antiporter (KcNHX1) mediated Na^+^ compartmentalization into the leaf vacuoles to maintain a low osmotic potential in the cytosol, avoiding Na^+^ toxicity under salt stress (Liu et al., 2012). Further, the plasma membrane Na^+^/H^+^ antiporter (KcSOS1) is a key systemic regulator of Na^+^ secretion by salt glands; it maintains K^+^/Na^+^ homeostasis via modulating the Na^+^ transport and K^+^ uptake/transporter system (Guo et al., 2020b). Other adaptive mechanisms of *K. caspia* to high‐salt environment need to be explored, particularly the possible roles of PM and SM changes. These would be especially relevant when salt crystals are deposited on the leaf surface from salt glands, as this would trigger leaf dehydration inducing intracellular oxidative damage. However, leaves of *K. caspia* do not show any sign of dehydration, possibly due to de novo synthesis of organic osmolytes facilitating the osmotic cellular adjustment (Shabala et al., 2014). Moreover, *K. caspia*, as a traditional Chinese medicinal herb, contained bioactive compounds, for example, phenylpropanoid glycosides, triterpenoids, sterols, and flavonoids (He et al., 2016; Yang et al., 2019b), implying that these could be potential ROS scavengers to be contributed to salt tolerance.

Therefore, we predicted that coordinated changes in PM and SM are important in the ability of *K. caspia* to cope with salt stress. In this current study, we identify differentially accumulated metabolites (DAMs), which relate to differentially expressed genes (DEGs) in leaves. Our results confirmed that changes in PM (starch and sucrose metabolism, TCA cycle, glycolysis, arginine and proline metabolism) and SM (chlorogenic acid‐ and vitamin E biosynthesis) contribute to the osmotic adjustment and ROS scavenging taking place in *K. caspia* exposed to salinity stress. These findings could be important for the exploitation of *K. caspia* in saline soil remediation.

## 2 MATERIALS AND METHODS

### 2.1 Plant materials and growth conditions

A single accession of *Karelinia caspia* (designated Guo1) was used as described by Guo et al. (2020b). The seeds were sown on black plastic tray containing John Innes No. 1 compost and sand (v/v, 4:1) under a daily photoperiod of 16/8 h (light/dark) at 24/20°C, relative humidity of 50–60% and 300–400 μmol m^−2^ s^−1^ photosynthetically active radiation in a growth chamber (Conviron, UK). The seedlings were selected for uniformity after 2 weeks, they were then transferred into plastic pots and irrigated every 2 days with tap water for 3 weeks. Our previous study showed that *K. caspia* plants grow well on 200 mM NaCl compared with control plants (Guo et al., 2020b); therefore, 5‐week‐old seedlings were watered with 0 or 200 mM NaCl for 2 days. All seedlings were grown in the same growth chamber. The pots were arranged in a randomized block design. After a 2‐day exposure period, shoots were harvested and washed thoroughly with tap water followed by deionized water. Samples were then oven‐dried at 80°C to a constant dry weight (DW). The relative growth rate (RGR) was calculated using the formula RGR = (ln*Wj* − ln*Wi*)/Δ*t*, where *Wj* and *Wi* are final and initial total DW, respectively, and Δ*t* is the time elapsed (days) between the two measurement (Martínez et al., 2005).

### 2.2 Measurements of chlorophyll fluorescence, imaging, and feature extraction

Chlorophyll fluorescence was performed at room temperature using a Handy‐PEA (Hansatech Instruments). Leaf surfaces were dark‐adapted for 30 min using dark leaf clips before measurements. After dark adaptation, the maximum quantum efficiency of PSII photochemistry (*F*_*v*_/*F*_*m*_) was instantly measured on leaf surface using a photosynthetic photon flux density of 3000 μmol m^−2^ s^−1^ as saturating flash for the duration of 1 s, where *F*_*v*_ was the difference between *F*_*m*_ and *F*_0_ (Sharma et al., 2015). For imaging and feature extraction, we captured the images before and after treatment using a camera, and exported them as RGB color images. Subsequently, the images were processed to extract features data including height and pixel values (green) according to C++ and OpenCV 2.4.9, an open‐source computer vision library (Fisher et al., 2016).

### 2.3 Measurements of water potential, osmotic potential, and turgor pressure in leaf

The PSYPRO water potential system (C‐52 Sample Chamber, WESCOR Inc.) was used to measure the leaf water potential (Ψ*w*, MPa) based on the manufacturer's instructions. Leaf osmotic potential (Ψ*s*) measurements were performed using the method described by Ma et al. (2012). In brief, fresh leaf samples were quickly frozen in liquid nitrogen and slowly thawed. A syringe was used to collect cell sap that was subsequently centrifuged at 9000*g* for 5 min. The osmolality (*n*, mmol kg^−1^) of the supernatant was recorded using a freezing point osmometer (Osmomat3000, Gonotec GmbH) at room temperature (25°C). The readings (*n*) were used to calculate the Ψ*s* (MPa) with the van't Hoff equation as ‐nRT, where *R* is the gas constant (0.008314 m^3^ MPa mol^−1^ K^−1^) and T is the thermodynamic temperature (298.8 K). Leaf turgor pressure (Ψ*t*, MPa) was calculated using the equation: Ψ*t* = Ψ*w* − Ψ*s* (Cui et al., 2020).

### 2.4 Determination of chlorophyll, lipid peroxidation, hydrogen peroxide, and antioxidant enzymes

Five hundred milligrams of fresh leaves was ground to powder in liquid nitrogen with a mortar and pestle. Analyses of leaf chlorophyll (Chl), malondialdehyde (MDA), hydrogen peroxide (H_2_O_2_) and the activity of superoxide dismutase (SOD, EC1.15.1.1) and peroxidase (POD, EC 1.11.1.7) were performed according to the methods in Guo et al. (2017). The activity of catalase (CAT, EC 1.11.1.6) was analyzed by measuring the residual H_2_O_2_ based on the method of Liao et al. (2019) and recorded at 240 nm every 1 min during 5 min. All optical density of reaction supernatant was measured using a UV2600 spectrophotometer (Techcomp). Each treatment was replicated six times, with two pooled plants per replicate.

### 2.5 Determination of cations concentration

Dried leaves and roots were ground and digested in a mixture of HNO_3_/HClO_4_ (5/1, v/v) for 24 h and then heated at 150–200°C to near dryness. After cooling, the residue was dissolved in distilled deionized water to a total volume of 25 ml. Na^+^, K^+^, and Ca^2+^ concentrations were determined using an atomic absorption spectrophotometer (AA‐6300C, Shimadzu).

### 2.6 RNA isolation, library construction, and sequencing

Total RNA was extracted from six samples using a plant RNA purification reagent (Invitrogen) according to the manufacturer's instructions. DNase I (Invitrogen) was added, and incubated at 37°C for 15 min to remove genomic DNA contamination. The concentration and quality of purified RNA was measured using the NanoDrop2000 Spectrophotometer (Thermo Scientific) and the Bioanalyzer 2100 system (Agilent Technologies), respectively. 1 μg of total RNA was used to construct a RNA sequencing (RNA‐seq) library using TruSeq RNA Sample Prep Kit according to the manufacturer's instructions (Illumina). The samples were sequenced on the Illumina HiSeq 2500 platform at Shanghai Majorbio Bio‐pharm Technology Co., Ltd.

### 2.7 De novo assembly and functional annotation

Raw reads were assessed by quality score using the Fastx_Toolkit (http://hannonlab.cshl.edu/fastx_toolkit/, version 0.0.14). Raw reads were trimmed and low‐quality reads removed using Seqprep (https://github.com/jstjohn/SeqPrep) and Sickle (https://github.-com/najoshi/sickle). The raw data has been deposited in the National Center for Biotechnology Information (NCBI) Sequence Read Archive under the accession number PRJNA744046.

De novo assembly of the clean reads was done using the paired‐end method of Trinity (https://github.com/trinityrnaseq/trinityrnaseq), and the derivation of unigenes was performed according to the method described by Blande et al. (2017). For gene functional annotations, all of the unigenes were aligned to six protein databases, Nr (NCBI nonredundant protein), Swiss‐Prot (Swiss‐Prot protein database), Pfam (Protein family), GO (Gene Ontology terms), COGs (Clusters of Orthologous Groups), and KEGG (Kyoto Encyclopedia of Genes and Genomes), using the BLASTX algorithm, with an *E* value threshold of 10^−5^ (Dong et al., 2014).

### 2.8 Differentially expressed genes (DEGs) and enrichment analysis

Gene expression was calculated by mapping clean reads to the unigenes database using the RSEM software package (Li & Dewey, 2011). The TPM (transcripts per million reads) method was used to normalize and quantify the expression levels of unigenes (Xue et al., 2010). The analysis of DEGs was performed by DESeq2 software under the standard parameter of the *p* value <0.05 and |log_2_ (FC)| >1 (Love et al., 2014). The BH (FDR correction with Benjamini/Hochberg) method was used to adjust *p* value. The identified DEGs were uploaded into the Majorbiol Cloud Platform (https://cloud.majorbio.com/) to analyze the expression differences, and GO terms/KEGG pathway enrichment according to FDR <0.05.

### 2.9 qRT‐PCR validation of candidate DGEs expression

Total RNA was extracted from leaves as described above. cDNA synthesis was conducted using a ×5 Primescript RTase mix (Takara, Biotech Co.). Quantitative real‐time RT‐PCR (qRT‐PCR) was performed on a thermal cycler (CF96X, Bio‐Rad) with the primers described in Table S1 and SYBR Green PCR (Takara Biotech Co.) as a master mix. *KcACTIN* was used as a reference gene for RNA normalization and was amplified in parallel with the target genes (Zhang et al., 2020). Relative expression levels were derived based on the 2^−ΔΔCt^ method (Guo et al., 2020b). Each experiment was carried out on three biological replicates.

### 2.10 Extraction and profiling of metabolites

The metabolite was extracted from leaves sample according to Paes de Araújo et al. (2019) with minor modifications. Briefly, approximately 50 mg of leaf was harvested in 2 ml microcentrifuge tube, and immediately frozen in liquid nitrogen. The frozen sample was ground into a powder with a sterile stainless steel ball for 30 s at 30 Hz using the oscillating mill MM200 (Retsch), then returned to the ice. 1 ml of prechilled chloroform: methanol: water (v/v/v, 1: 2.5: 1) was added into each sample, vortexed, then shaken at 4°C for 20 min, followed by microcentrifuging at 21,000 *g* for 6 min at 4°C. 100 μl of supernatant was transferred into a glass vial and capped for subsequent flow injection electrospray ionization high resolution‐mass spectrometry (FIE‐HRMS). Each treatment was performed six times (one independent leaf sample in each replicate).

FIE‐HRMS was performed using Q‐Exactive plus mass analyzer instrument with ultra‐high pressure liquid chromatography system (Thermo Fisher Scientific), where mass‐to‐charge ratios (*m*/*z*) were generated in positive (POS) and negative (NEG) ionization mode in a single run as described by Beckmann et al. (2008) and Baptista et al. (2018). The scanning ranges were from 55–280 to 270–1200 in both POS and NEG ionization mode. Signals were normalized to total ion count. All metabolomics data assessed using MetaboAnalyst (version 5.0) software (Pang et al., 2021). In brief, data were filtered by interquantile range, log_10_ transformed and Pareto scaled. Student *t*‐test was used to identify the significant *m*/*z*, which was uploaded into the specific module (MS peaks to pathways) to match potential metabolites using the mummichog algorithm and interrogation of KEGG and MZedDB (http://maltese.dbs.aber.ac.uk:8888/hrmet/index.html) databases based on 5 ppm thresholds and considering various ionization forms of metabolite feature. Correlation analyses, PCA, hierarchical cluster analyses and debiased sparse partial correlation (DSPC) analyses were performed using the R‐based platform MetaboAnalyst 5.0 (Pang et al., 2021).

### 2.11 Statistical analysis

For the physiological parameters analyses, each treatment was repeated six times, each replicates consisting of a pool of three plants. Data are presented as means with SD. Data analyses, including one‐way ANOVA and Duncan's multiple range tests, were performed by SPSS version 23.0 software (IBM SPSS statistics, SPSS Inc.).

## 3 RESULTS

### 3.1 Assessing the phenotypic, physiological, and biochemical responses to salt in *K. caspia*

**FIGURE 1:**
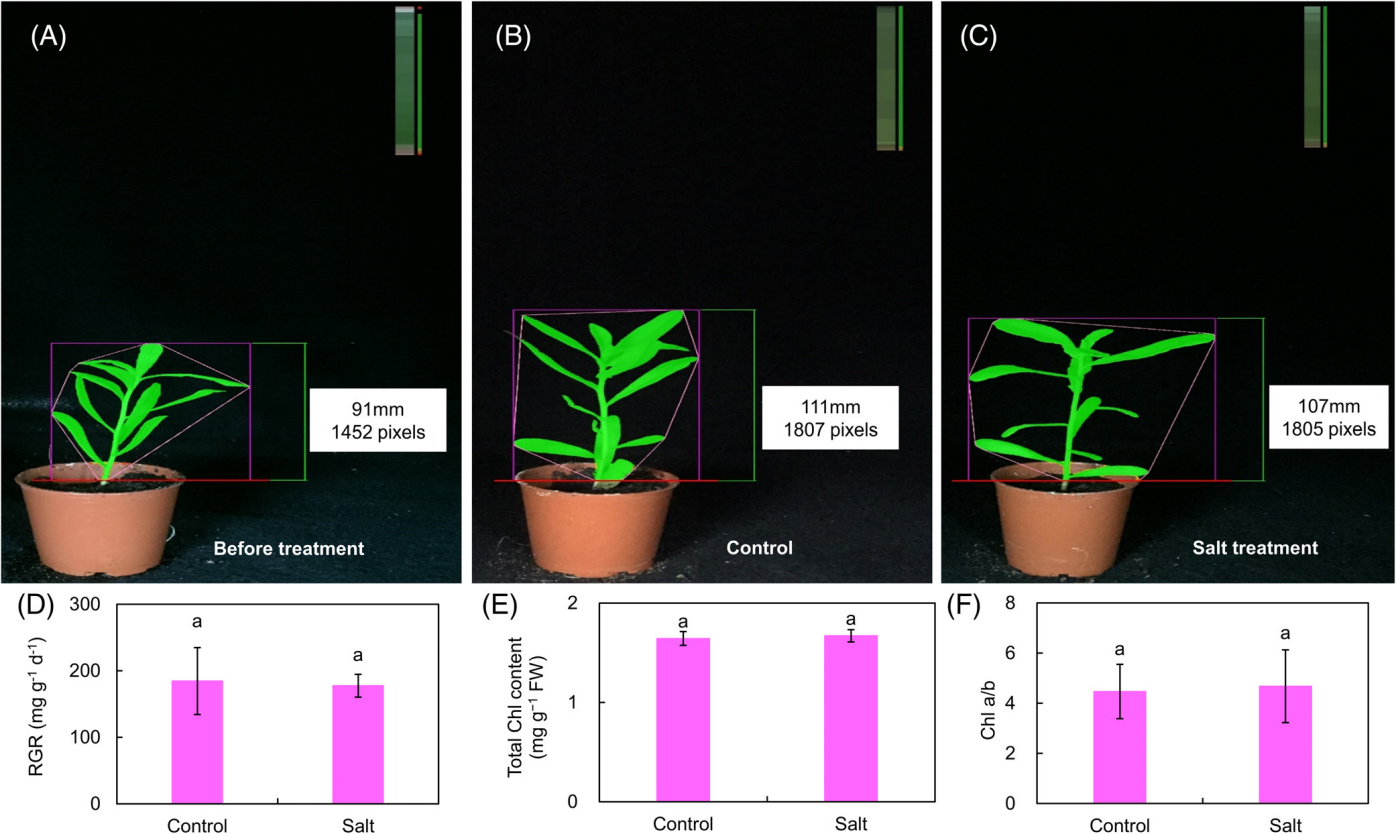
(A–C) *Karelinia caspia* phenotypic features (height and color) before treatment, and after 2 days at the control or salt treatment. Each pixel number reflects the side projective area of each plant and therefore increases with plant biomass. The color bars show the color distribution of the plants. The control and salt treatment plants have higher green color percentage. (D) Analyses of relative growth rate (RGR), (E) total chlorophyll (Chl) content, and (F) Chl *a*/*b* in *K. caspia* seedlings under control and salt conditions. Data are means ± SD (*n* = 6 independent plants). Different letters indicate significant differences at *p* <0.05 (Duncan's test)

The impact of salt stress on the growth and photosynthesis parameters was determined in *K. caspia* in response to control (0 mM NaCl) and salt (200 mM NaCl) treatments after 2 days. Compared with the control, salt treatment had no impact on the phenotypic features (height and color) based on RGB image analyses (Figure 1A–C). No marked differences were observed in relative growth rate (RGR) between control and salt (Figure 1D). The absence of effects was also seen in total Chl content, Chl *a*/*b*, chlorophyll fluorescence parameters (Figure 1E–F and Table S2). These data confirmed that *K. caspia* exhibits good salt tolerance.

To further examine the salt‐induced oxidative stress in *K. caspia*, the accumulation of the membrane lipid peroxidation biomarker malondialdehyde (MDA) as well as H_2_O_2_ and antioxidant enzyme activities were assessed. There were no significant differences in MDA or H_2_O_2_ content between the leaves of the control and salt‐treated plants (Table 1). However, the addition of 200 mM NaCl increased the activities of SOD, POD, or CAT, which were 1.43‐, 1.88‐, or 1.77 fold higher, respectively, than that of controls (Table 1). This suggests that enhanced antioxidant activities protect against the salt‐induced oxidative stress.

**TABLE 1:** Effect of salt treatment on lipid peroxidation, H_2_O_2_ accumulation, and the activity of SOD, POD, and CAT in the leaves of *K. caspia*

NaCl treatment (mM)	MDA content (μmol g^−1^ FW)	H_2_O_2_ content (μmol g^−1^ FW)	SOD activity (U g^−1^ FW)	POD activity (U g^−1^ min^−1^ FW)	CAT activity (U g^−1^ min^−1^ FW)
0	0.21 ± 0.058a	0.77 ± 0.07a	60.28 ± 7.16b	16.67 ± 3.72b	125 ± 28.87b
200	0.24 ± 0.042a	0.81 ± 0.087a	86.07 ± 7.64a	31.33 ± 6.89a	221.33 ± 46.33a

*Note*: Data are means ± SD (*n* = 6 independent plants). Different letters indicate significant differences at *p* < 0.05 (Duncan's test).

### 3.2 Ions accumulation and osmotic adjustment in salt‐treated *K. caspia*

**FIGURE 2:**
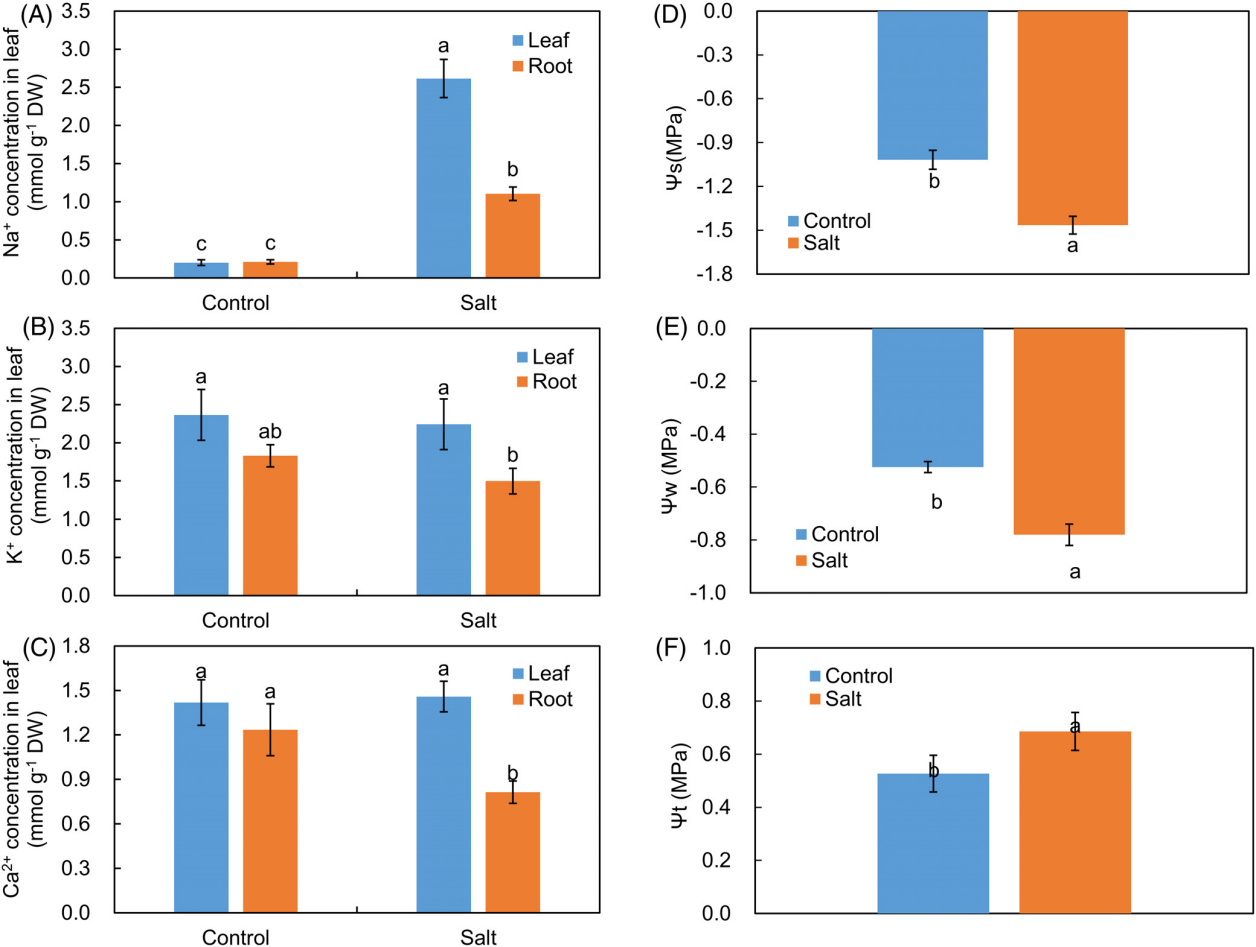
Effect of the control and salt treatment on tissue's Na^+^ (A), K^+^ (B), and Ca^2+^ (C) concentration and (D) leaf osmotic potential (Ψ*s*), (*E*) leaf water potential (Ψ*w*), and (*F*) leaf turgor pressure (Ψ*t*) in *K. caspia*. Data are means ± SD (*n* = 6, independent plants). Different letters indicate significant differences at *p* <0.05 (Duncan's test)

To assess the impact of salt stress on induced ionic imbalance, Na^+^, K^+^, and Ca^2+^ concentrations were measured in *K. caspia* submitted to salt treatment for 2 days. Under the control conditions, there was no difference in Na^+^, K^+^, or Ca^2+^ concentrations between leaf and root (Figure 2). Conversely, Na^+^ concentrations were greatly increased in both leaves and roots under salt treatments relative to the controls (Figure 2A). However, no significant differences were found in K^+^ or Ca^2+^ in leaves between the controls and salt treatments (Figure 2B,C), indicating that *K. caspia* could maintain a constant K^+^ and Ca^2+^ concentrations under salt stress.

We then estimated the effect of salt on osmotic adjustments within the leaves of *K. caspia* (Figure 2D–F). Salt treatment markedly decreased leaf Ψ*w* and Ψ*s*, but significantly enhanced leaf Ψ*t* in comparison with the controls. These data demonstrated that *K. caspia* could accumulate osmolytes, including inorganic salts and organic solutes (metabolites), to improve the hydration and turgor pressure in the leaf.

### 3.3 Metabolomic analysis in the leaf of *K. caspia* response to salt stress

**FIGURE 3:**
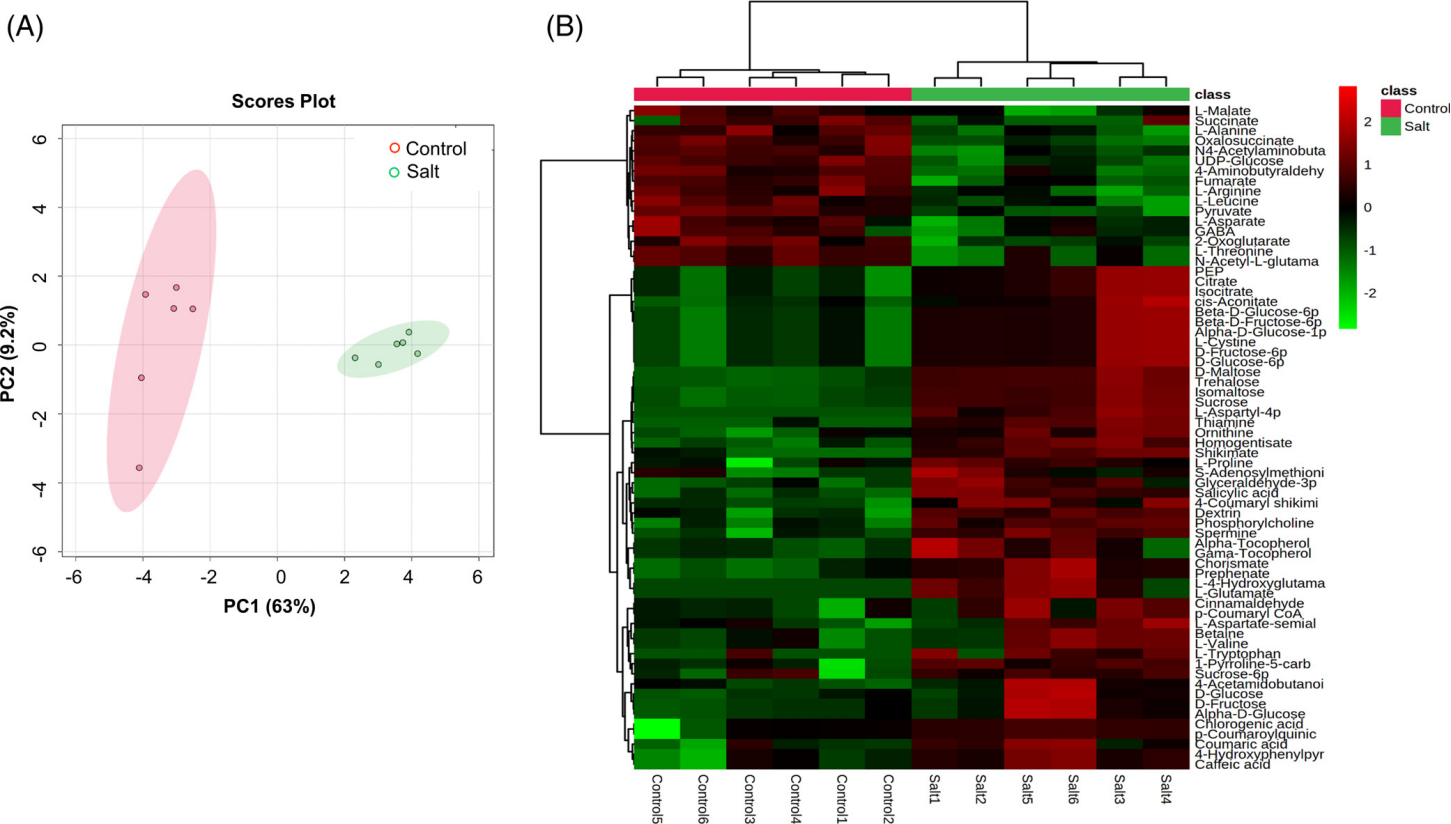
Metabolomics analysis of leaves in *K. caspia* exposed to control and salt treatments. (A) Principal component analysis (PCA) of 66 metabolites detected by FIE‐HRMS. (B) Heatmap for hierarchical clustering analysis of differentially accumulated metabolites (DAMs) associated with primary and secondary metabolism. The color scale indicates the different log_10_ (FC) values of DAMs

Metabolomics assessments were based on the use of FIE‐HRMS. The derived data were assessed using PCA, which showed a clear separation between control and salt‐treated *K. caspia* leaves with PC1 explaining 63% of the total variation (Figure 3A). The major sources of variation were further determined using *t*‐tests and corrected for false discovery rates (FDR). A total of 66 DAMs were subsequently identified based on accurate mass, isotope and ionization patterns. Most DAMs were primary metabolites (i.e., amino acids, amines, organic acids, and carbohydrates) except a plant hormone (salicylic acid, SA), vitamins (α‐tocopherol, γ‐tocopherol, and thiamine) and some secondary metabolites (betaine, caffeic acid, chlorogenic acid, *p*‐coumaric acid, *p*‐coumary CoA, 4‐coumaryl shikimic acid, and *p*‐coumaryl quinic acid). When displayed using a heatmap, 50 and 16 DAMs were up‐ and downregulated under salt stress, respectively, compared with the control group (Figure 3B).

**FIGURE 4:**
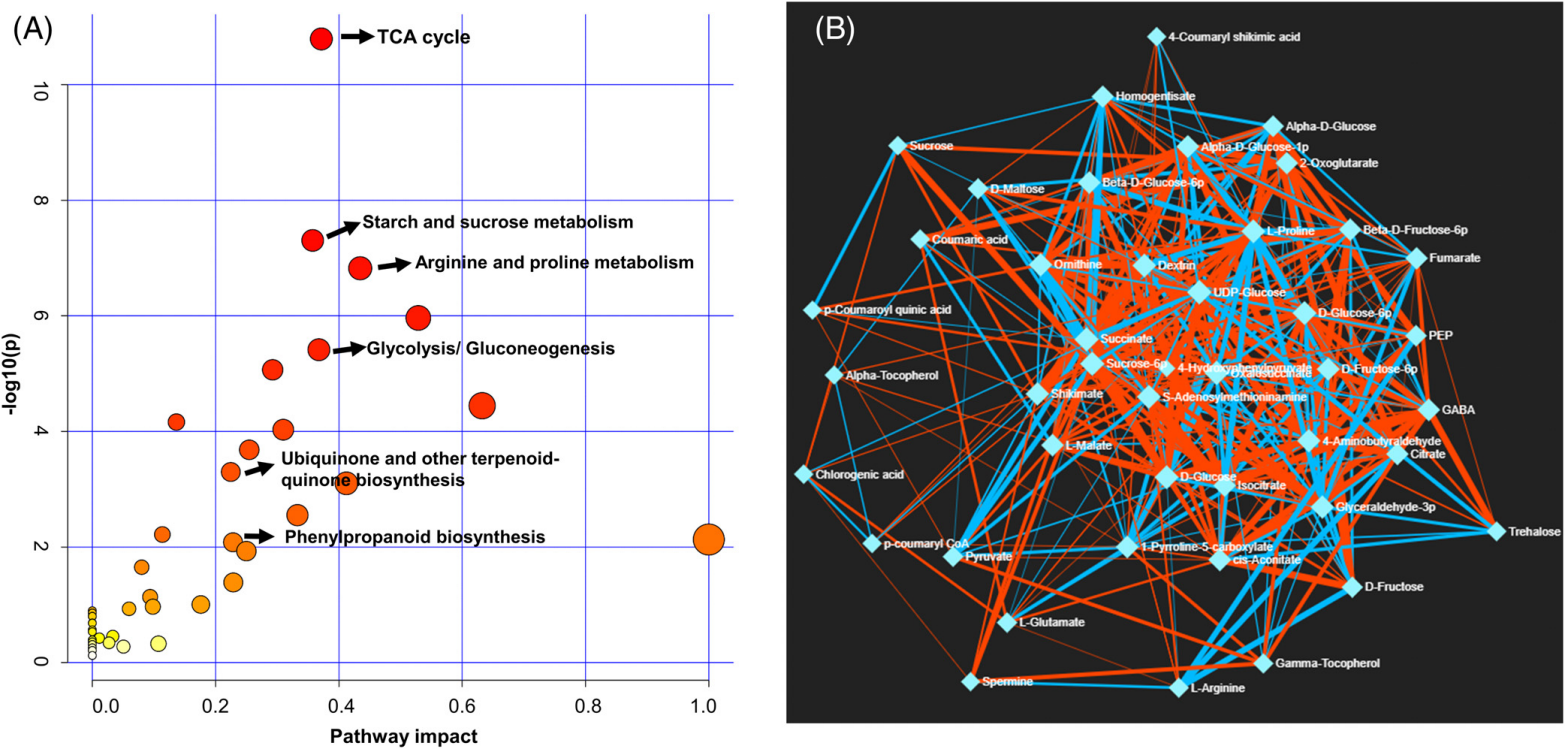
Metabolic pathways analysis in *K. caspia* exposed to the control and salt treatment. (A) KEGG pathway analysis based on DAMs. Every circle represents a metabolic pathway, with red or yellow color indicating high or low impact. (B) The interaction relationships between DAMs was analyzed by debiased sparse partial correlation (DSPC) network. Nodes represent metabolites, and edges indicate the correlation between metabolites. Nodes are colored to correspond to their compound classes

To investigate the metabolic pathways involved in *K. caspia* exposed to salt stress, the DAMs were mapped to the Arabidopsis KEGG pathway library. DAMs could be mapped to the following metabolic pathways: citrate cycle (TCA cycle), starch and sucrose metabolism, arginine and proline metabolism, glycolysis/gluconeogenesis, ubiquinone and other terpenoid‐quinone biosynthesis, as well as phenylpropanoid biosynthesis based on an FDR <0.05 and impact value >0.2 (Figure 4A). Moreover, there were significant correlations between these DAMs as indicated by debiased sparse partial correlation (DSPC) network analysis (Figure 4B). For example, UDP‐glucose was correlated with dextrin, D‐glucose 6‐phosphate, 4‐hydroxyphenylpyruvate, sucrose 6‐phosphate, and succinate, implicating UDP‐glucose is an important precursor of these metabolites when *K. caspia* is subjected to salt stress.

### 3.4 RNA‐seq, de novo assembly and functional annotation in the leaf of *K. caspia* under salt stress

To compare these changes with the salt‐responsive transcriptome in *K. caspia*, cDNA libraries were constructed from leaves of plants grown in the absence or presence of 200 mM NaCl for 2 days. The libraries were sequenced using the Illumina HiSeq 2500 platform. Control samples generated from 44.3‐ to 51.5 million clear reads, while 42.2‐ to 44.8 million clear reads were obtained insalt‐treated samples, with Q20 over 98%, Q30 over 95%, and GC content over 45% (Table S3). This indicated that the RNA sequencing output and quality were adequate for further analysis.

A total of 42,678 unigenes were subsequently assembled from the short clean reads using Trinity software. The average unigenes length was 1049.67 bp, 16,772 unigenes had a length over 1000 bp (Table S4). More than 45% of the unigenes were annotated from public protein libraries (NR, SwissProt, Pfam, COG and GO) with a cut‐off *E* value of 10^−5^ (Table S5). The remainder could not be annotated as a *K. caspia* reference genome has yet to be generated.

Next, unigenes expression levels and differentially expressed genes (DEGs) between the control and salt‐treated groups were defined. Using RSEM software, the unigene expression distribution had a mean maximum and minimum of 4.85 and 1.22 for controls and 4.82 and 0.95 for salt treatment based on counting the transcripts per million (TPM) (Figure S1A). The Venn diagram showed that 19,695 unigenes were co‐expressed between the control and salt stress groups (Figure S1B). In line with this, Pearson correlation analysis also indicated some similarity in transcriptome profiles (*r* ^2^ = 0.727 to 0.989) between the three replicates of the two sample groups (Figure S1C). Nevertheless, PCA analysis could distinguish between the samples without or with salt treatment (Figure S2A). DEGs were identified between the control and salt‐treated groups using DESeq2, with a *p* value <0.05 and log_2_ (FC)| >1. The volcano plots showed that 2290 or 1939 DEGs were upregulated or downregulated by salt treatment compared with the control group (Figure S2B); they are displayed using a heatmap (Figure S2C).

To further gain insight into the functions of the DEGs underlying salt tolerance, these were linked to GO terms and assessed by KEGG enrichment analyses using the goatools platform. GO annotation analysis indicated that DEGs were significantly enriched in biological process (BP), cellular component (CC), and molecular function (MF) (Figure S2D). Additionally, KEGG enrichment analyses were conducted to assess the association between DEGs and metabolic pathways in *K. caspia* under salt stress. KEGG enrichment analyses categorized 224 DEGs into 20 pathways. These were related to PM metabolism (amino acids and carbohydrates), plant hormone signal transduction, MAPK signaling, biosynthesis of secondary metabolites, and carbohydrate metabolism (Figure S2E).

### 3.5 Correlation analyses of the expression of metabolism‐related genes and corresponding metabolites in the leaf of *K. caspia*

**FIGURE 5:**
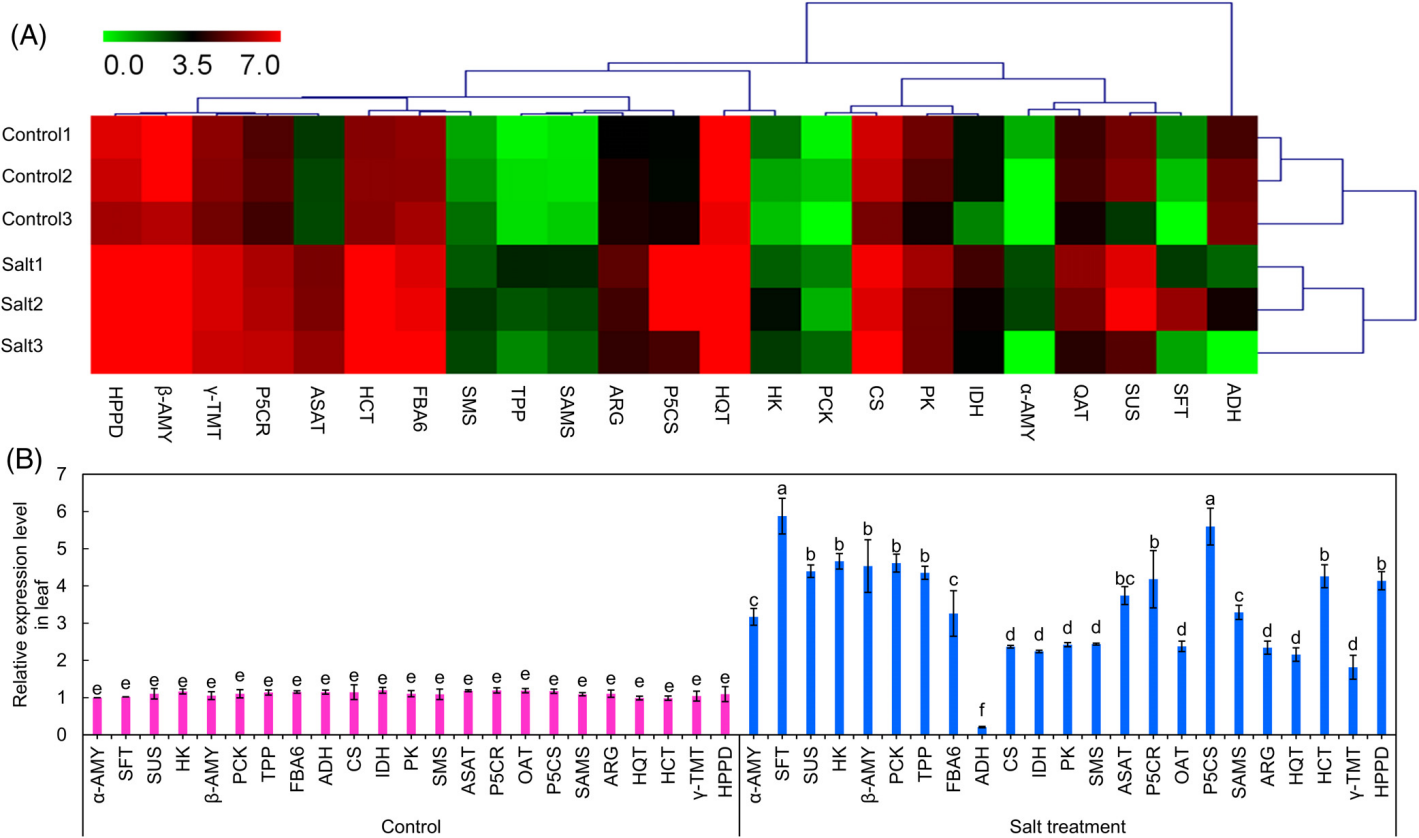
Analysis of leaf gene expression profiles in the representative metabolic pathways in *K. caspia* exposed to the control and salt treatment. (A) Hierarchical cluster analysis was performed on data acquired by RNA‐seq. Heat map was constructed for TPM of DGEs associated with TCA cycle, starch and sucrose metabolism, arginine and proline metabolism, glycolysis/gluconeogenesis, ubiquinone and other terpenoid‐quinone biosynthesis, as well as phenylpropanoid biosynthesis. The color scale represents the upregulated or downregulated genes expression based on the thresholds of *p* value <0.05 and |log_2_ (FC)| >1. (B) Validation of the expression of the candidate DEGs using qRT‐PCR. Data are means ± SD (*n* = 3 independent samples). Different letters indicate significant differences at *p* <0.05 (Duncan's test)

To further explore the salt mechanisms of metabolic responses in *K. caspia* at the transcriptome level, we mapped the expression level of genes related to KEGG pathways including carbohydrate metabolism (starch and sucrose metabolism, glycolysis and TCA cycle), arginine, and proline metabolism as well as chlorogenic acid/vitamin E biosynthesis. From the transcriptome data and the KEGG pathway assessment, we focused on genes involved in carbohydrate metabolism that were upregulated by salt compared with controls (Figure 5A): *HK* (*hexokinase*, TRINITY_DN12312_c0_g1), *SFT* (*sucrose 1‐fructosyl transferase*, TRINITY_DN9570_c0_g1), *TPP* (*trehalose 6‐phosphate phosphatase*, TRINITY_DN8280_c1_g1), *SUS* (*sucrose synthase*, TRINITY_DN8807_c0_g1), *α‐AMY* (*α‐amylase 1*, TRINITY_DN28863_c0_g1), *β‐AMY* (*β‐amylase 1*, TRINITY_DN9956_c0_g2), *FBA6* (*fructose‐bisphosphate aldolase 6*, TRINITY_DN123_c0_g1), *PCK* (*phosphoenolpyruvate carboxykinase*, TRINITY_DN8290_c0_g1), *CS* (*citrate synthase*, TRINITY_DN4762_c0_g1), *IDH* (*isocitrate dehydrogenase*, TRINITY_DN31728_c0_g1), and *PK* (*pyruvate kinase*, TRINITY_DN1926_c0_g1). Also, key enzymes related to arginine and proline metabolism were all upregulated by salt: *SMS* (*spermine synthase*, TRINITY_DN2782_c0_g4), *SAMS* (*s‐adenosylmethionine synthase*, TRINITY_DN29693_c0_g1), *OAT* (*ornithine aminotransferase*, TRINITY_DN16267_c0_g1), *ARG* (*arginase*, TRINITY_DN9521_c0_g1), *P5CR* (*pyrroline‐5‐carboxylate reductase*, TRINITY_DN16336_c0_g2), *ASAT* (*aspartate aminotransferase*, TRINITY_DN4614_c0_g3), as well as *P5CS* (*delta‐1‐pyrroline‐5‐carboxylate synthase*, TRINITY_DN14855_c0_g1). Of the genes examined, only *ADH* (*aldehyde dehydrogenase*, TRINITY_DN1085_c0_g1) was downregulated (Figure 5A). Furthermore, salt stress elevated the expression level of *HQT* (hydroxycinnamoyl‐CoA: quinate hydroxycinnamoyl transferase, TRINITY_DN7710_c0_g2) /*HCT* (*hydroxycinnamoyl‐CoA: shikimate/quinate hydroxycinnamoyl transferase*, TRINITY_DN6971_c0_g1) and HPPD (hydroxyphenyl pyruvate dioxygenase, TRINITY_DN6847_c0_g2)/γ‐TMT (TRINITY_DN14107_c1_g1) involved in the biosynthesis of phenylpropanoid (chlorogenic acid) or ubiquinone and other terpenoid‐quinone (vitamin E). These results were all validated by qRT‐PCR (Figure 5B).

**FIGURE 6:**
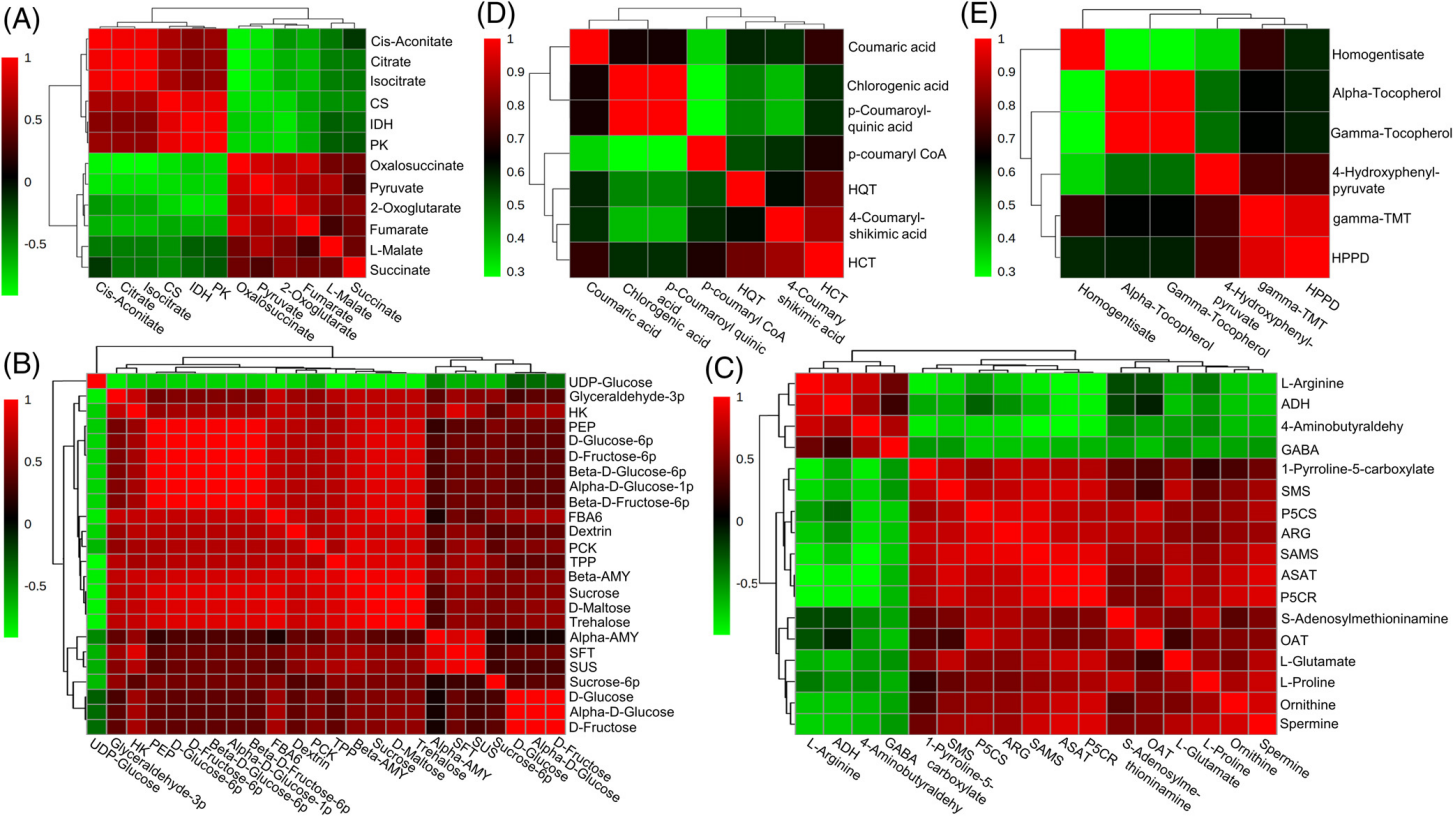
Correlation analysis between metabolome and transcriptome in the leaf of *K. caspia* exposed to the control and salt treatment. The heat map shows the correlation coefficient between DEGs and DAMs involved in (A) TCA cycle, (B) starch and sucrose metabolism plus glycolysis/gluconeogenesis, (C) arginine and proline metabolism, (D) phenylpropanoid (chlorogenic acid) biosynthesis, as well as (E) ubiquinone and other terpenoid‐quinone (vitamin E) biosynthesis by Pearson's correlation analyses

**FIGURE 7:**
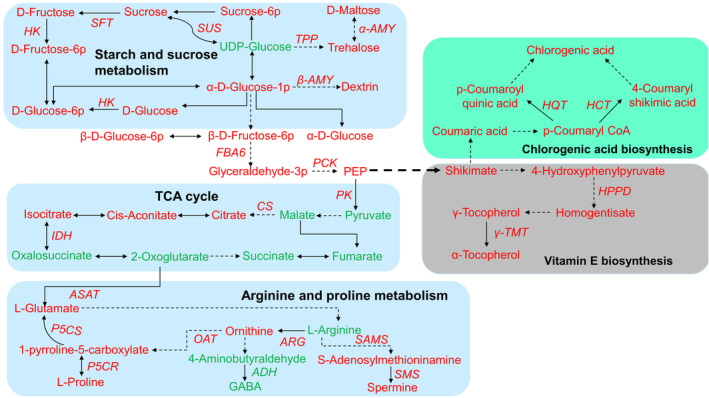
Schematic diagram of the changes in expression level of key genes and corresponding metabolites mapped into representative metabolic pathways (starch and sucrose metabolism, glycolysis, TCA cycle, arginine and proline metabolism, chlorogenic acid/vitamin E biosynthesis) in leaf of *K. caspia* exposed to salt. Upright or italics represents metabolites or key enzyme genes. Red or green color shows upregulation or downregulation, respectively, in metabolites and genes

As expected, the modulation in the expression of these genes correlated with the corresponding metabolite contents (Figure 6). Using the DSPC tool, the changes in enzymes‐encoding genes and their corresponding metabolites are visualized (Figure 7), where upregualtions are shown in red and downregualtion in green compared with controls. These data suggested that carbohydrate metabolism (starch and sucrose metabolism plus TCA cycle), proline/spermine biosynthesis, as well as chlorogenic acid/vitamin E biosynthesis are involved in the salt response of *K. caspia*.

## 4 DISCUSSION

*Karelinia caspia* is a forage species and traditional Chinese medicine, which occupies an important place in the North China desert ecosystem by being very salt‐tolerant and involved in stabilizing and potentially remediating saline soils. Beyond its Chinese context, it could represent a model system to study the mechanisms of salt tolerance in halophytes. This knowledge could then be used to inform crop breeding programs. To define the mechanisms of salt tolerance in *K. caspia*, we employed transcriptomic and metabolomic approaches.

### 4.1 Saccharide metabolism and proline/spermine biosynthesis facilitate the osmotic adjustment in *K. caspia* under salt stress

Salt stress led to low osmotic potential and high turgor pressure in *K. caspia* leaves (Figure 2). We suggest that the accumulation of organic osmolytes (saccharides, proline, and spermine) and inorganic ions (Figures 2 and 3) facilitated the continued water influx and turgor maintenance under salt conditions (Cui et al., 2020).

Saccharides' metabolism is involved in modulating many physiological processes, including responses to abiotic stresses in plants (Chen et al., 2019). Our data showed that salt treatment upregulated the sucrose content and transcription of *SUCROSE SYNTHASE* (*SUS*) as well as a slight increase in chlorophyll content (Figure 6B and Table S2), suggesting that *SUS* could maintain photosynthesis even in stress conditions as already suggested (Li et al., 2021). We found an increased transcription of *SUCROSE 1‐FRUCTOSYL TRANSFERASE* (*SFT*) and fructose concentrations with salt stress (Figure 6B), implying sucrose was converted into fructose to be involved in saccharides metabolism via *SFT*. Meanwhile, the upregulation of *HEXOKINASE* (*HK*) was significantly correlated with the accumulation of glucose, glucose‐6p, fructose, and fructose‐6p in our gene‐metabolite interaction network (Figure 6B). This reflected the role of hexokinase in hexose phosphorylation to regulate saccharide utilization, hence serving as a sensor for glycolysis of D‐glucose and D‐fructose (Li et al., 2018). In addition, sucrose synthase converts sucrose into UDP‐glucose (UDPG) (Gao et al., 2020) to be further catalyzed into trehalose‐6‐phosphate (T6P) under trehalose 6‐phosphate synthase, followed by T6P was dephosphorylated to trehalose by TPP (Iordachescu & Imai, 2008). In line with this, the transcriptional and metabolite components of sucrose flux to trehalose were significantly activated upon salt treatment in our study (Figure 7). This agreed with the role of trehalose as an established compatible solute in osmotic adjustment and protein/membrane protectant under abiotic stress (Elbein et al., 2003).

Proline is an organic osmolyte that has an important osmoprotective role in cells and also enhances the ability of antioxidant enzymes to remove ROS during salt stress (Ghahremani et al., 2014). It seems likely that the biosynthesis and accumulation of proline are affected in *K. caspia* by salt as we observed salt‐upregulated *ASAT*/*P5CS*/*OAT*/*P5CR* expression and the corresponding metabolites (glutamate/1‐pyrroline‐5‐carboxylate/ornithine/proline) (Figure 6C). 2‐oxoglutarate is an important precursor of the biosynthesis of glutamate via aspartate aminotransferase (Singh et al., 2016). Glutamate is reduced to L‐glutamate‐5‐semialdehyde by delta‐1‐pyrroline‐5‐carboxylate synthetase, which is further converted into 1‐pyrroline‐5‐carboxylate (P5C) (Hu et al., 1992; Szabados & Savoure, 2010). Ornithine is also catalyzed into P5C by ornithine aminotransferase, and then it is reduced to proline in the cytosol by pyrroline‐5‐carboxylate reductase (Anwar et al., 2018). Given these observations, we propose that this route is mainly responsible for biosynthesis and accumulation of proline in the response of *K. caspia* to salt stress.

**FIGURE 8:**
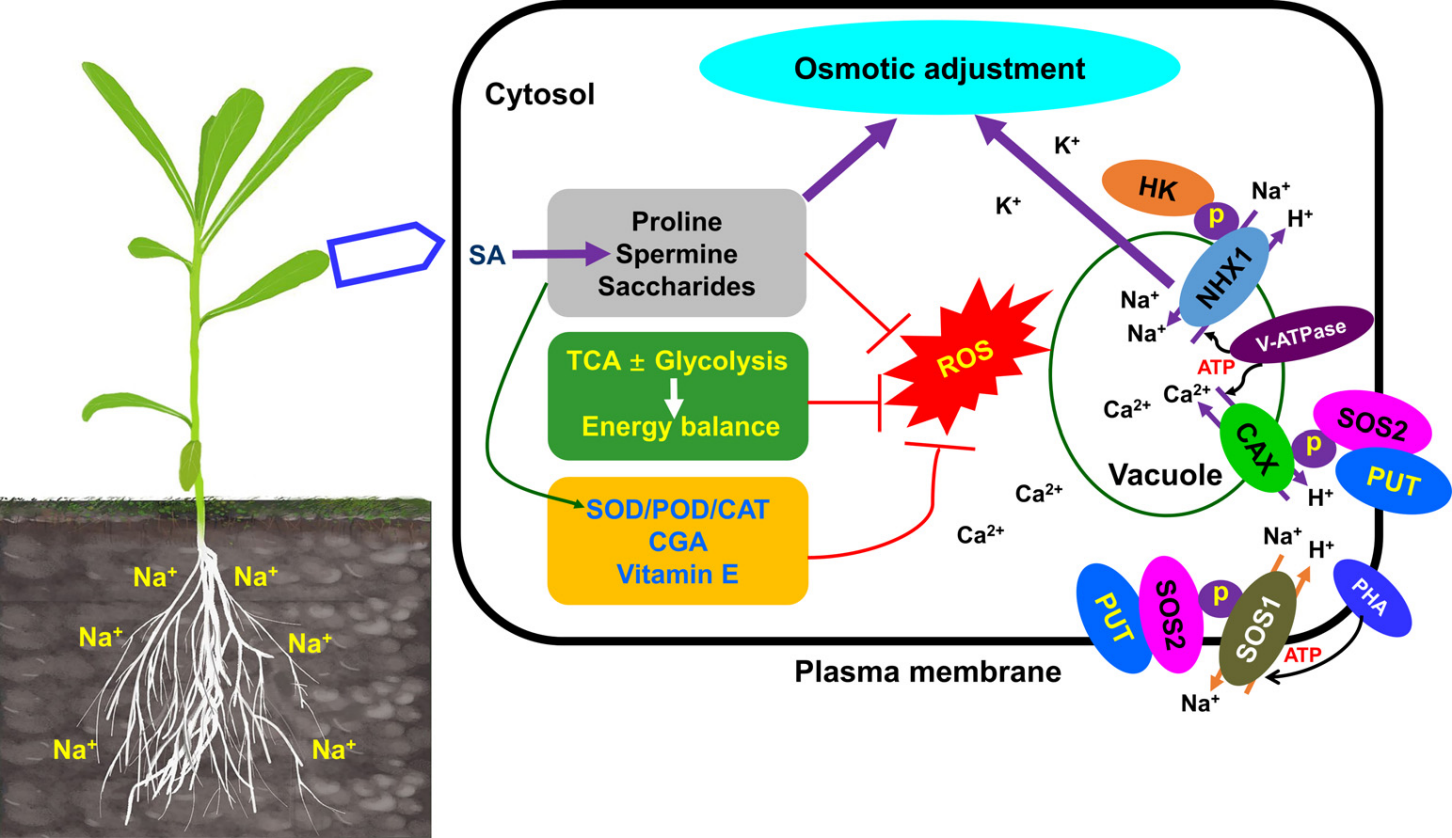
Schematic model of osmotic adjustment, ROS scavenging, and ions homeostasis in leaf cells of *K. caspia* treated with salt. In the presence of salt, (1) organic osmolytes, such as proline, spermine, and saccharides increase dramatically. HK phosphorylated NHX1 to promote the sequestration of cytoplasmic Na^+^ into vacuoles. Both organic osmolytes and inorganic ions contribute to osmotic adjustment to reduce cellular osmotic potential and improve turgor pressure. (2) Accumulation of organic osmolytes and elevated salicylic acid could indirectly enhance the antioxidant enzyme activities (SOD/POD/CAT) to reduce the excess ROS. The accumulation of CGA and vitamin E will also scavenge ROS. In addition to this, low TCA cycle flux may counteract glycolysis‐associated energy consumption to suppress ROS overproduction. (3) PUT might be involved in recruiting and activating SOS2 at the plasma membrane to facilitate the transport activity of SOS1 and CAX to extrude Na^+^ into the apoplast and sequestrate Ca^2+^ into vacuoles, respectively, thereby modulating ions homeostasis

Polyamines (PAs) are small aliphatic nitrogenous bases with two or more amino groups that include putrescine, spermidine, and spermine and are widely distributed in plant species (Liu et al., 2020). In PA biosynthesis, arginine/ornithine can be catalyzed to putrescine by arginine decarboxylase/ornithine decarboxylase. Subsequently, spermidine and spermine are synthesized by sequential additions of aminopropyl groups to putrescine and spermidine, respectively, by the concerted action of spermidine synthase/s‐adenosylmethiomine decarboxylase and spermine synthase (Sánchez‐Rodríguez et al., 2016). In our study, we showed that salt stress upregulated the expression of *SAMS* and *SMS* and the corresponding metabolites: s‐adenosylmethiomine and spermine, respectively (Figure 6C). These data suggested that the spermine biosynthesis route could be dependent on the substrate s‐adenosylmethiomine and related to PA biosynthetic enzymes. The accumulation of spermine is regarded as an important indicator of salt tolerance in plants (Maiale et al., 2004), where it acts as a signaling molecule leading to the activation of antioxidant enzymes (Liu et al., 2015; Seifi & Shelp, 2019). Spermine would therefore contribute to the ROS homeostasis in salt‐treated *K. caspia* by improving the activity of antioxidant enzymes (Table 1). Elevated spermine is also regarded as one of the most effective osmoprotectants against salt stress (Paul & Roychoudhury, 2016). This would align with the low osmotic potential and high turgor pressure in salt‐treated *K. caspia* leaves, which appeared to be associated with the accumulation of organic osmolytes (saccharides/proline/spermine) (Figures 2D–F and 3). The increased SA hints in its roleas an important regulator of salt responses in *K. caspia* (Figure 3). SA can increase polyamine content under salt stress and this has been linked to increased salt tolerance in tomato (Szepesi et al., 2009) and *Medicago sativa* (Palma et al., 2013). SA can also increase Na^+^ levels in leaves as well as suppressing ROS (Gémes et al., 2011). The roles of SA in salt responses in recretohalophytes have not been extensively explored, although one report suggests that SA down‐regulation in *Aeluropus lagopoides* was important for salt tolerance (Paidi et al., 2017). This did not tally with the increases seen upon salt treatment in *K. caspia*, further work is needed to assess the role of SA in this species. To aid to the comprehension, the proposed role of saccharide metabolism and proline/spermine biosynthesis in *K. caspia* subjected to salt is summarized in Figure 8.

### 4.2 Role of TCA cycle in modulating energy balance in *K. caspia* under salt stress

Increased respiratory generation of energy is a survival strategy for plants to adapt to various stressful environments (Chen et al., 2019). Energy production requires the coordination of several metabolic processes, including glycolysis and the oxidative pentose phosphate pathway in the cytoplasm/plastid and the mitochondrial TCA cycle (Bandehagh & Taylor, 2020). The increase in the glycolysis metabolites glyceraldehyde‐3p, PEP and associated gene expression with salt treatment (Figure 6A) suggests that elevated bioenergetic metabolism was also a feature of the salt response in *K. caspia*. However, this need not be a beneficial feature as it could be linked to the enhanced production of ROS (Tiwari et al., 2002). Thus, enhancing the accumulation of glycolysis metabolites could be coupled with the generation of ROS in *K. caspia*.

Glycolysis feeds into the TCA cycle to drive ATP synthesis via the oxidation of respiratory substrates (Sweetlove et al., 2010). Elevated TCA cycle activity has been shown to improve the salt tolerance of wild soybean and Kentucky bluegrass by increasing the energy capacity and the levels of intermediate products under alkaline‐salt stress (Hu et al., 2014; Li et al., 2017). However, other literatures have suggested that the concentrations of TCA‐cycle intermediates are lower with salt stress, for example, in maize hybrids (Richter et al., 2015). Indeed, the salt‐sensitive genotype of Tibetan wild barley (XZ169) consumed more energy in the TCA cycle than the tolerant genotype (XZ26) (Shen et al., 2016). Such discrepancies suggest that the role of the TCA cycle in responses to salt stress should be considered separately in each species. In *K. caspia*, a reduction in most of the intermediates associated with the TCA cycle was observed under salt stress, including fumarate, malate, oxalosuccinate, 2‐oxoglutarate, pyruvate, and succinate (Figure 3). Such changes could indicate that *K. caspia* adopts an energy conservation strategy, where there is a transition from plant growth to the induction of protective mechanisms and protein synthesis under salt stress (Liu & Howell, 2010), although this does not appear to be reflected in our measurements of RGR (Figure 1D). Another important clue could be that high salinity had no impact on H_2_O_2_ content compared with the unstressed plants (Table 1). Therefore, the low level of TCA cycle flux may partially counteract glycolysis‐associated ROS overproduction in responses of *K. caspia* to salt (Bandehagh & Taylor, 2020). However, it should be noted that TCA metabolism associated with citrate accumulation was actually increased in *K. caspia* (Figure 3). Citrate accumulation has been linked to the maintenance of pH homeostasis in *Puccinellia tenuiflora* under alkaline stress (Shi et al., 2002); it might be playing a similar role in salt tolerance in *K. caspia*. These hypothesized roles are included in Figure 8.

### 4.3 The contribution of hexokinase and polyamine transporter to ions homeostasis in *K. caspia* under salt stress

Hexokinase is a sensor for the glycolysis of D‐glucose to modulate saccharides utilization in planta (Li et al., 2018). We observed that salt stress upregulated *HEXOKINASE* and glucose in leaves of *K. caspia* (Figure 6B). Interestingly, the apple glucose sensor MdHXK1 (HEXOKINASE1) could interact with and phosphorylate MdNHX1 (Na^+^/H^+^ EXCHANGER 1) to transport the intracellular Na^+^ into vacuoles to maintain the ion homeostasis under salt stress (Sun et al., 2018). Previous studies showed that the expression of *KcNHX1* was induced and regulated by salt stress, which is responsible for sequestering Na^+^ into leaf vacuoles (Guo et al., 2020b; Liu et al., 2012). If the precedent established with the apple glucose sensor MdHXK1 is valid for *K. caspia* (Sun et al., 2018), we speculate that the hexokinase‐NHX1 regulatory module might contribute to maintain Na^+^ homeostasis in *K. caspia* in response to salt. In addition to this, salt‐treated plants could enhance the level of spermine to alter Ca^2+^ allocation to modulate Ca^2+^ homeostasis (Yamaguchi et al., 2006). The SALT OVERLY SENSITIVE 2 (SOS2) protein kinase also specifically activates the tonoplast Ca^2+^/H^+^ antiporter CAX1 that is involved in Ca^2+^ homeostasis under salt stress (Cheng et al., 2004). Such observations suggest that polyamines (PAs), especially spermine, might have regulatory links between protein kinase and ion transporters in plants subjected to salt stress. Furthermore, our data showed that salt upregulated the transcription of *PUT* (plasma membrane polyamine transporter gene)/*CAX*/*SOS1/SOS2* (data not shown) and *SRM*, accompanied by the increase in spermine and Na^+^ concentration but no impact on the concentrations of K^+^ and Ca^2+^ in leaves (Figures 2A and 6C). Notably, a recent report in *Arabidopsis* indicated that the PUT3‐SOS1‐SOS2 complex could not only modulate SOS1‐mediated Na^+^ efflux to reduce cytosolic Na^+^ concentrations but also activate PUT3 to promote the accumulation of PAs to protect cells from oxidative damage (Chai et al., 2020). Our previous findings showed that KcSOS1 participated in the process of Na^+^ secretion to maintain K^+^/Na^+^ homeostasis at a whole plant level (Guo et al., 2020b). Based on such evidence, we cannot rule out that PUT might recruit and activate SOS2 at the plasma membrane to facilitate the transport activity of SOS1 and CAX, further modulating ions homeostasis in *K. caspia* under salt conditions (Figure 8). However, these hypotheses will need to be confirmed by further research.

### 4.4 Chlorogenic acid/vitamin E biosynthesis may be involved in the maintenance of ROS homoeostasis in *K. caspia* under salt stress

Chlorogenic acid (CGA) is an antioxidant phenolic acid that can aid the prevention of cardiovascular disease and other age‐related diseases (Meinhart et al., 2019). The CGA biosynthesis pathway is defined in different plant species (Clifford et al., 2017). Besides the better‐established route based on shikimate, another pathway is based on hydroxycinnamoyl‐CoA: quinate hydroxycinnamoyl transferases (HQT) prefers to use quinate as an acyl acceptor (Kriegshauser et al., 2021). Suppressing HQT leads to a dramatic reduction in CGA content (by 98%) in *Nicotiana benthamiana*, demonstrating that HQT is directly involved in CGA biosynthesis (Niggeweg et al., 2004; Sonnante et al., 2010). Other literature also proved that HQT2 is the last rate‐limiting enzyme controlling the production of CGA in *Taraxacum antungense* (Liu et al., 2019). In line with such reports, we observed that the upregulation of *HQT* expression correlated with the accumulation of CGA and its precursor in *K. caspia* subjected to salt stress (Figure 6D). In addition to this, increases in AtHQT orthologous hydroxycinnamoyl‐CoA, shikimate/quinate hydroxycinnamoyl transferases (AtHCT), were noted. HCT also has a broad acceptor substrate range (Hoffmann et al., 2003; Sander & Petersen, 2011) and has been mainly linked to lignin biosynthesis and composition (Hoffmann et al., 2004). However, HCT2 has also been associated with CGA biosynthesis in *Cecropia obtusifolia* and *Populus*. In the latter, HCT2 is involved in the defense response via the WRKY transcriptional regulatory pathway (Cadena‐Zamudio et al., 2020; Zhang et al., 2018). Based on these observations, we suggest that HQT or HCT is a key enzyme that may recognize an acyl acceptor, quinic acid or shikimic acid to determine CGA biosynthesis in salt‐treated *K. caspia* (Figure 7). Meanwhile, the accumulation of CGA could be linked to the maintenance of redox homeostasis under salt stress (Kiani et al., 2021). As our data showed that ROS levels are unchanged in *K. caspia* exposed to salt (Table 1), it may be fulfilling a similar role here.

Vitamin E, a potent lipid‐soluble antioxidant protecting cell membranes from free radical damage is also a relevant compound of the oxidative homeostasis (Muñoz & Munné‐Bosch, 2019). We observed the up‐regulation upon salt treatment of a key gene *HPPD* (*HYDROXYPHENYL PYRUVATE DIOXYGENASE*) involved in vitamin E biosynthesis as well as the content of important intermediates of the biosynthetic pathway: 4‐hydroxyphenylpyruvate (HPP) and homogentisate (HGA) (Figure 6E). HPP derives from tyrosine degradation (Riewe et al., 2012) and is then converted into HGA by the enzymatic action of HPPD (Mène‐Saffrané & Pellaud, 2017). HGA and phytyl diphosphate (PDP) are condensed into the intermediate methylphytyl benzoquinone (MPBQ) by the homogentisate phytyl transferase (HPT) enzyme (Chen et al., 2006). HPT is one of the most important enzymes catalyzing the later steps of the vitamin E biosynthetic pathway along with γ‐TMT (Shintani & DellaPenna, 1998). In line with this, the expression of *γ‐TMT* was positively correlated with the accumulation of γ‐tocopherol in *K. caspia* under salt stress (Figure 6E), implying its importance in the salt response. This was supported by recent research showing that co‐overexpression of *AtHPT* and *Atγ‐TMT* enhances the α‐tocopherols content and cellular antioxidant enzyme activity, hence improving salt and cadmium tolerance in potato (Upadhyaya et al., 2021). The likely important role of the end product α‐tocopherol content in vitamin E biosynthetic pathway in conferring protection from oxidative damage in *K. caspia* under salt stress is included in Figure 8.

## 5 CONCLUSIONS

Our present study demonstrated that the adaptation of *K. caspia* to soil salinity may be modulated at transcriptomic and metabolomic levels. Some of these primary metabolites (saccharides, TCA intermediates, proline, and spermine) and key secondary metabolites (CGA and vitamin E) were closely associated with the transcriptional levels of genes coding for enzymes participating in fundamental biological processes during salinity stress. Notably, at transcriptional and metabolic levels, the key genes and metabolites linked to saccharide metabolism, TCA cycle, proline/spermine biosynthesis and CGA/vitamin E biosynthesis might facilitate the osmotic adjustment and remove the excess ROS in *K. caspia* under salt conditions. Therefore, our findings provide novel insights for understanding the inter‐coordination of multiple metabolic pathways that contribute to the adaption of *K. caspia* to saline environments.

## AUTHOR CONTRIBUTIONS

Luis A. J. Mur, Juying Wu, and Qiang Guo conceived and designed this research. Qiang Guo, Jiwan Han, Cui Li, Chunqiao Zhao, Qinghai Wang, and Xincun Hou performed the experiments. Qiang Guo and Jiwan Han carried out data analysis. Qiang Guo wrote the manuscript. Luis A. J. Mur reviewed and edited the manuscript. We declare no conflicts of interest in relation to this study.

## Supporting information


**Appendix S1**: Supporting information

## Data Availability

The data that support the findings of this study are openly available in NCBI Sequence Read Archive database (accession number: PRJNA744046).
