# Supplementary material for: Defining key metabolic roles in osmotic adjustment and ROS homeostasis in the recretohalophyte Karelinia caspia under salt stress
Source: Physiol Plant. 2022 Mar 14;174(2):e13663. doi: 10.1111/ppl.13663 (PMC9311275; doi:10.1111/ppl.13663)
Supplement: Supplementary file 1 — Appendix S1: Supporting information [file PPL-174-0-s001.pdf]

Defining key metabolic roles in osmotic adjustment and ROS homeostasis in the recretohalophyte *Karelinia caspia* under salt stress

Qiang Guo, Jiwan Han, Cui Li, Xincun Hou, Chunqiao Zhao, Qinghai Wang, Juying Wu, Luis A.J. Mur

Table S1. Primer sequences used in the experiments

Primer	Sequence (5'-3')
HK-F	5'-TCACCTTCTCATTTCCCGTGTTGC-3'
HK-R	5'-CCCTTTCCTTTCCATCGCTTTTGC-3'
SFT-F	5'-AACGCACTGAGCAACTTCCTGTC-3'
SFT-R	5'-ACTGCTTCCGTAAACCCATTTCCC-3'
TPP-F	5'-TGTTGGGGTTGAGCTTGCTTCTG-3'
TPP-R	5'-ACTTGAGGGCTGATGGATACTGGAG-3'
SUS-F	5'-TGGCTAACCTTGTAGTCGTTGCTG-3'
SUS-R	5'-GGGCTGAAATCCATCTTACCTGACC-3'
α-AMY-F	5'-TTGGCACAGTTATCCGCAAGTGG-3'
α-AMY-R	5'-TGATCCTCCAGCACCATGTCCTC-3'
β-AMY-F	5'-TGTATAGAGATGCGGGACCACGAG-3'
β-AMY-R	5'-CCTGTGCTTCCTGGGTTGCTAAC-3'
FBA6-F	5'-CTCAATACTATGCCGCTGGTGCTC-3'
FBA6-R	5'-TGGCTAACTGCGATGGCTCATTG-3'
PCK-F	5'-GTGACGGACCAGACCTCTTTGAAC-3'
PCK-R	5'-ATCAACGGAGTGAAGATCGAAGCG-3'
CS-F	5'-GAGTGCCCTTTCTGTATTCCATCCC-3'
CS-R	5′-CCTGCCATCCTTAAGTAAGCTGCTG-3'
IDH-F	5'-TTCATGGTTCGGCTCCTGATATTGC-3'
IDH-R	5'-TGGCGAAGCATTGATACAGCACTC-3'
PK -F	5'-GCTTAGCCTCATCCGCAGTTCG-3'

PK -R	5'-AGGAACCACCACCGAGAGAATAGG-3'
SMS -F	5'-GCCGCCTGGAGACTTCAAGATTC-3'
SMS -R	5'-GACAAATGAAGGCAACGCGAAGG-3'
SAMS -F	5'-TGTAACGCCGGAAGATGGTTTCAG-3'
SAMS -R	5'-CACCGCCTTCTTCACCATGTCAG-3'
OAT -F	5'- TGGAAGAATGCTGGCATGTGACTG -3'
OAT -R	5'- ATGACTCCACCACCCAATGCTTTAC -3'
ARG -F	5'- TGCATTTGCTCCACCAAGGATACG -3'
ARG -R	5'- CCACAGTCTCGAAGCTCTTGAACAG -3'
P5CR -F	5'-CTGGTGTCAGTAGTTGCTGGTGTC-3'
P5CR -R	5'-AGCAGGCGTATTTGGCATTACCC-3'
ASAT-F	5'-TGCTTCAATCGTTGCTACCATCCTC-3'
ASAT-R	5'-TGGCTCCAATCTCCAGGTGTCC-3'
P5CS -F	5'-AGCTTCATTGGCAATCCGTAGTGG-3'
P5CS -R	5'-GTCAGTGAGGGCAGAAGTGATTACC-3'
ADH-F	5'-TCCTCAGTTCTCGCTAACACAATCC-3'
ADH-R	5'-CCAACATCGGGTCCACCTTCAAC-3'
HQT-F	5'-CATGTCGAGTACCACCTGCCTCC-3'
HQT-R	5'-GTTGGTGTTTCCGCCATCATTC-3'
HCT-F	5'-GGAGGAGTGGGTCTTGGTTGTGG-3'
HCT-R	5'-AATGAAAGGTGGAATGGCTACGG-3'
γ-TMT-F	5'-TTTTATCTTCCTGCTTGGTGTT-3'
γ-TMT-R	5'-AATAGTCTTCCATCCGCTCTTTA-3'
HPPD-F	5'-CCACTGCTTCCATCCCGACCTT-3'
HPPD-R	5'-GTGGGCGACACTGACGGAGAAA-3'
ACTIN-F	5'-CTTGCGTATGTGGCTCTTGACT-3'
ACTIN-R	5'-TGGAACAAAACCTCTGGACAAC-3'

Table S2. Effect of salt treatment on the maximal quantum yield of PSII photochemistry (Fv/Fm), the actual PSII efficiency (Φ_{PSII}), photochemical quenching coefficient (*q*P), and non-photochemical quenching coefficient (*q*NP) in the leaf of *K. caspia*. Data are means ± SD (n = 6). Different letters indicate significant differences at P<0.05 (Duncan's test).

NaCl treatment (mM)	Fv/Fm	Φ_{PSII}	*q*P	*q*NP
0	0.78±0.007a	0.38±0.023a	0.5±0.025a	0.3±0.059a
200	0.79±0.03a	0.39±0.019a	0.52±0.022a	0.32±0.025a

Table S3. Output statistics of RNA-sequencing.

Sample	Raw reads	Raw bases	Clean reads	Clean bases	Error rate (%)	Q20 (%)	GC content (%)
control_1	44329704	6693785304	43968138	6604871420	0.0236	98.65	45.96
control_2	49759056	7513617456	49332524	7409666834	0.0236	98.65	45.85
control_3	51547716	7783705116	51173512	7694421778	0.0237	98.62	46.09
salt_1	44810802	6766431102	44354590	6671567762	0.0235	98.67	45.75
salt_2	44649844	6742126444	44329290	6669562475	0.0236	98.66	45.85
salt_3	42207828	6373382028	41734422	6276474087	0.0234	98.69	49.7

Table S4. Summary of de novo sequence assembly.

Total unigenes number	Total length (bp)	Average length (bp)	N50 length (bp)	GC percent (%)	Unigenes number (> 1000 bp)
42678	44797604	1049.67	1666	40.28	16772

Table S5. Information of unigenes annotation.

Database	GO	KEGG	COG	NR	Swiss-Prot	Pfam	Total annotation
Hit numbers	21224	11749	22726	24852	19458	19815	25719
(%)	(49.73%)	(27.53)	(53.25)	(58.23)	(45.59)	(46.43)	(60.26)

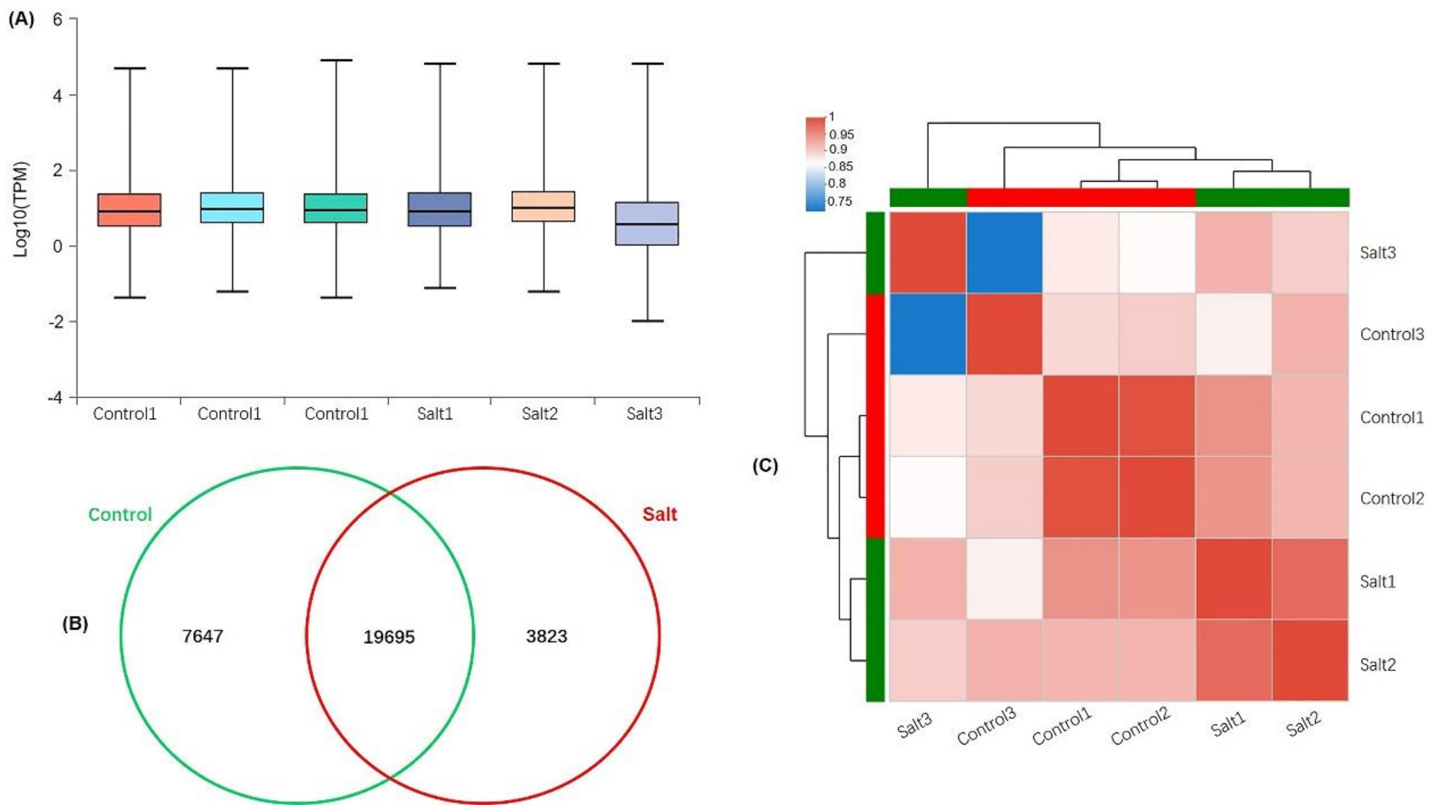

Fig. S1 Summary of unigenes expression between control- and salt treatment group. (A) Distribution of unigenes expression values (B) Venn diagram of co-expressed and specifically expressed unigenes, (C) correlation analysis of unigenes expression between two sample groups.

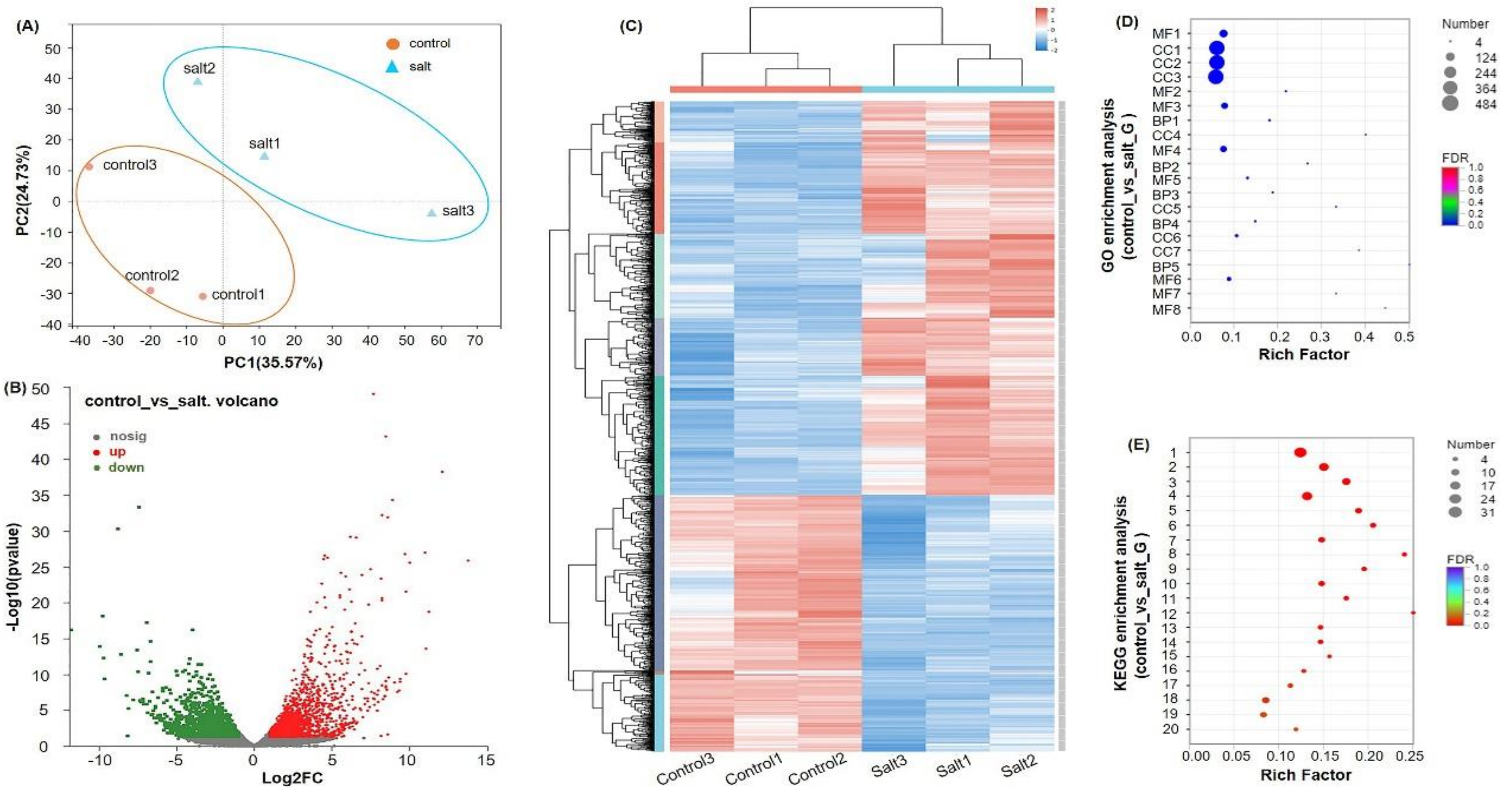

Fig. S2 Transcriptome analysis in leaf of *K. caspia* exposed to the control and salt treatment. (A) PCA score plot; (B) Volcano plot of DEGs. The dots with red or green color represent the upregulated or downregulated genes based on the thresholds of p-value < 0.05 and $|\log_2 (FC)| >1$; (C) Heat map for cluster analysis of the DEGs. The color scale indicates the different $\log_{10}$ (TPM) values of DEGs; (D) GO- and (E) KEGG enrichment

analysis of DEGs. Top 20 GO terms showed are MF1(oxidoreductase activity), CC1 (integral component of membrane), CC2 (intrinsic component of membrane), CC3 (membrane part), MF2 (oxidoreductase activity, acting on paired donors, with oxidation of a pair of donors resulting in the reduction of molecular oxygen to two molecules of water), MF3 (transmembrane transporter activity), BP1 (amine metabolic process), CC4 (plastid nucleoid), MF4 (transporter activity), BP2 (hormone metabolic process), MF5 (dioxygenase activity), BP3 (response to water), CC5 (nucleoid), BP4 (lipid transport), CC6 (extracellular region), CC7 (chloroplast nucleoid), BP5 (proline metabolic process), MF6 (active transmembrane transporter activity), MF7 (galactosyltransferase activity), MF8 (UDP-glucose 4-epimerase activity), respectively. Top 20 KEGG pathway indicated are plant hormone signal transduction (1), cysteine and methionine metabolism (2), glycine, serine and threonine metabolism (3), MAPK signaling pathway-plant (4), biosynthesis of unsaturated fatty acids (5), zeatin biosynthesis (6), arginine and proline metabolism (7), tropane, piperidine and pyridine alkaloid biosynthesis (8), phenylalanine metabolism (9), tyrosine metabolism (10), alpha-Linolenic acid metabolism (11), diterpenoid biosynthesis (12), flavonoid biosynthesis (13), galactose metabolism (14), isoquinoline alkaloid biosynthesis (15), beta-Alanine metabolism (16), alanine, aspartate and glutamate metabolism (17), starch and sucrose metabolism (18), phenylpropanoid biosynthesis (19), steroid biosynthesis (20), respectively. Numbers above each bar represents DEG numbers. The color scale indicates rich factor of DEGs.
